# Supplementary material for: Association between handgrip strength, handgrip strength asymmetry, and anxiety in Korean older adults: The Korean National Health and Nutrition Examination Survey 2022
Source: PLoS One. 2025 Apr 8;20(4):e0315256. doi: 10.1371/journal.pone.0315256 (PMC11978050; doi:10.1371/journal.pone.0315256)
Supplement: Attachment — Submitted filename: Response to Reviewers_sangyoun_choi.docx [file pone.0315256.s002.docx]

**Revision Note for Manuscript ID PONE-D-24-53685**

**Association between handgrip strength, handgrip strength asymmetry, and anxiety in Korean older adults: The Korean National Health and Nutrition Examination Survey 2022**

We appreciate the opportunity to revise our paper. When revising, we closely considered your comments and suggestions. As requested, we have explained the changes made in response to the reviewer's feedback. The reviewer's insight was extremely helpful, and we are grateful for constructive feedbacks on the initial submission. By addressing the raised points, we believe our paper's quality has significantly improved and we hope you share this view. Below, we provide our response to each comment, and a revision note is attached highlighting the amended sections of the manuscript. Once again, thank you for your valuable and helpful feedback.

**Response to Editorial Board Member’s comments**

Thank you for submitting your manuscript to PLOS ONE. After careful consideration, we feel that it has merit but does not fully meet PLOS ONE’s publication criteria as it currently stands. Therefore, we invite you to submit a revised version of the manuscript that addresses the points raised during the review process.

- *Thank you for your thorough review of our manuscript. We will carefully read your comments and thoughtfully consider your valuable suggestions. Following the review, we have completely revised our manuscript. Below is our response to each comment.*

Journal Requirements:

When submitting your revision, we need you to address these additional requirements.

1. Please ensure that your manuscript meets PLOS ONE's style requirements, including those for file naming. The PLOS ONE style templates can be found at

https://journals.plos.org/plosone/s/file?id=wjVg/PLOSOne_formatting_sample_main_body.pdf and

https://journals.plos.org/plosone/s/file?id=ba62/PLOSOne_formatting_sample_title_authors_affiliations.pdf

- *Thank you for pointing this out. The manuscript was carefully prepared to fully comply with PLOS ONE's style requirements, including proper formatting of the text and alignment with the style templates provided. No revisions were necessary, as the original submission already met these standards.*

2. Please include your full ethics statement in the ‘Methods’ section of your manuscript file. In your statement, please include the full name of the IRB or ethics committee who approved or waived your study, as well as whether or not you obtained informed written or verbal consent. If consent was waived for your study, please include this information in your statement as well.

- *Thank you for highlighting the importance of the ethics statement. The ‘Methods’ section of the manuscript already includes the complete ethics statement, specifying the full name of ethics committee that approved or waived the study. It also clarifies that written or verbal consent was obtained.*

3. Please amend either the title on the online submission form (via Edit Submission) or the title in the manuscript so that they are identical.

- *Thank you for bringing this to my attention. I have amended the title listed in the online submission form to ensure consistency with the title in the manuscript itself. Both titles now match exactly, addressing this requirement effectively.*

**Response to Reviewer #1’s comments**

This study explores the association between handgrip strength (HGS), HGS asymmetry, and anxiety among older adults in Korea, utilizing data from the 2022 Korea National Health and Nutrition Examination Survey (KNHANES). The finding of a significant correlation between low HGS and anxiety in women is particularly noteworthy. The research addresses an important public health topic and highlights the potential utility of HGS as a marker for mental health issues.

However, the study suffers from critical limitations, particularly the restricted sample size for subgroup analyses, insufficient depth in the interpretation of findings, and a lack of clarity on whether complex sampling analysis was conducted. Additionally, challenges in presenting data effectively further limit the study’s clarity and generalizability.

- *Thank you for your thorough review of our manuscript. We will carefully read your comments and thoughtfully consider your valuable suggestions. Following the review, we have completely revised our manuscript. Below is our response to each comment.*

**Point 1**

1. The data source, KNHANES, uses a complex sampling design involving stratification, clustering, and weighting to ensure population representativeness. However, the manuscript does not mention whether these design features were accounted for in the analysis.

A. Clarify whether the complex sampling design was incorporated into the analysis

B. Discuss the implications of not using complex sampling in the limitations section if it cannot be incorporated retroactively.

**Response 1:** *We thank for pointing this out. We have added explanation about the complex sampling design incorporated into the analysis.*

***Revised manuscript, line 126-128, page 7, Method: We used stratification, clustering, and weighting variables in the KNHANES data to ensure population representativeness. We used weighting variables that consider both health and nutrition factors.***

**Point 2**

2. Subgroup analyses (e.g., by education level or area of residence) are undermined by small sample sizes, leading to wide confidence intervals and a lack of statistical significance, even in cases where the effect size appears substantial. For example, in the "college or higher" education subgroup among women, a high odds ratio (OR) was reported, but it did not achieve statistical significance due to the limited sample size.

A. Aggregate or simplify subgroup categories where appropriate to strengthen the robustness of statistical analyses.

B. Discuss the impact of small sample sizes on the interpretation of results more explicitly in the limitations section.

**Response 2:** *We thank you for the comments. Following your suggestions, we attempted to simplify the subgroup categories. However, all the variables were necessary, and we could not aggregate the subgroup categories because each variable had only two categories, except for obesity, which had three. While we initially discussed this limitation in the discussion section, we realized that our explanation was insufficient. Therefore, we have elaborated more explicitly on the limitation of small sample sizes.*

***Revised manuscript, line 260-263, page 17, Discussion: Additionally, we identified more cases with high odds ratios that did not achieve statistical significance, including the rural group (aOR: 2.54, 95% CI: 0.92–6.98), the obese group (aOR: 2.45, 95% CI: 0.94–6.38), and the low-stress group (aOR: 4.83, 95% CI: 0.94–24.73).***

**Point 3**

3. The observed gender differences in the results, particularly the lack of significant findings in men, are not sufficiently explained.

A. Elaborate on the biological, social, or cultural factors that may account for the gender differences observed in the association between HGS and anxiety.

B. Include additional references to explain why men might be less affected by HGS changes in relation to anxiety compared to women.

**Response 3:** *We thank you for the comments. Initially, we attributed the difference in results to the disparity in average HGS between men and women. However, we found this explanation insufficient and identified the patriarchal mindset as an additional contributing factor.*

***Revised manuscript, line 230-239, page 16, Discussion: A patriarchal mindset prevalent among the current older Korean adults may also contribute to this difference. Having grown up in a patriarchal society, many of them hold the belief that men should earn money while women should handle housework. As a result, retired men often do not feel obligated to engage in any form of work, including domestic chores, whereas women experience pressure to manage household responsibilities. This dynamic means that women, rather than men, are more likely to feel anxious when low HGS prevents them from fulfilling their expected roles. Given that 49.4% of the male survey population was not working, the disparity between retired men and women may significantly amplify the impact of low HGS on women’s anxiety.***

**Point 4**

4. The presentation of data in tables can be improved to enhance readability.

A. Including both the odds ratio (OR) and the corresponding p-value in the results table is essential for a more complete and transparent understanding of the findings.

**Response 4:** *We thank you for the comments. We have added p-values to all the results tables that were previously missing them.*

***Revised manuscript, Table 2,3,4,5:***
