# Supplementary material for: Association between handgrip strength, handgrip strength asymmetry, and anxiety in Korean older adults: The Korean National Health and Nutrition Examination Survey 2022
Source: PLoS One. 2025 Apr 8;20(4):e0315256. doi: 10.1371/journal.pone.0315256 (PMC11978050; doi:10.1371/journal.pone.0315256)
Supplement: Attachment — Submitted filename: Response to Reviewers_sangyoun_choi_2.docx [file pone.0315256.s003.docx]

**Revision Note for Manuscript ID PONE-D-24-53685**

**Association between handgrip strength, handgrip strength asymmetry, and anxiety in Korean older adults: The Korean National Health and Nutrition Examination Survey 2022**

We are grateful for the chance to revise our paper. In doing so, we carefully addressed your comments and suggestions. As instructed, we have detailed the changes made in response to the reviewer’s feedback. Their insights were immensely valuable, and we sincerely appreciate the constructive input on our initial submission. By incorporating the points raised, we believe the quality of our paper has been greatly enhanced, and we hope you agree. Below, we have provided a response to each comment, along with a revision note that highlights the updated sections of the manuscript. Thank you once again for your thoughtful and helpful feedback.

**Response to Reviewer #1’s comments**

This manuscript presents an important study on the association between Handgrip Strength (HGS) and Anxiety. However, there are some concerns regarding the clarity of confounding variables and the lack of Crude OR (Unadjusted OR) in the results tables (Table 2, 3, 4, 5). These issues may reduce the reliability of the findings. To improve the manuscript, the following revisions are strongly recommended.

- *We sincerely appreciate the detailed review of our manuscript. We will thoroughly examine your comments and give careful consideration to your insightful suggestions. In response to your feedback, we have made comprehensive revisions to the manuscript. Below, we address each of your comments individually.*

**Point 1.** The "Statistical Analysis" section should clearly specify which variables were used as confounders. A detailed list of confounders (e.g., age, education level, marital status, obesity, drinking, sleep duration, stress level, physical activity, smoking, etc.) should be explicitly provided. It should also be clearly stated whether these variables were used only as control variables or analyzed as independent predictors of anxiety.

**Response 1:** *Thank you for your valuable feedback. We have revised the "Statistical Analysis" section to specify the confounders used in the analysis. As suggested, we have provided a detailed list of confounders, including age, education level, working state, residence, marital status, obesity, drinking, sleep duration, stress level, physical activity, and smoking. Additionally, we have clarified that these variables were included solely as control variables and were not analyzed as independent predictors of anxiety.*

***Revised manuscript, line 129-131, page 7, Methods: Age, education level, working state, residence, marital status, obesity, drinking, sleep duration, stress level, physical activity, and smoking were used as confounders. These variables were included solely as control variables and not as independent predictors of anxiety.***

**Point 2 & 3 (related to Table 2,3,4,5)**

**Point 2.** Currently, only Adjusted OR (aOR) is provided, making it difficult to evaluate the effect of confounders on the results. Crude OR (Unadjusted OR) should be included in the tables to allow a direct comparison with Adjusted OR. This will enhance the transparency of how confounding variables affect the observed associations.

**Response 2:** *Thank you for your valuable feedback regarding the presentation of the results. We agree that providing both Crude OR (Unadjusted OR, cOR) and Adjusted OR (aOR) in the tables would enhance transparency and allow for a clearer evaluation of the effect of confounders on the observed associations.*

*In response to your comment, we have revised Tables 2, 3, 4, and 5 to include both Crude OR and Adjusted OR. This modification allows for a direct comparison between unadjusted and adjusted estimates, providing a more comprehensive understanding of how confounding variables influence the results. We also added explanations in manuscript to clarity if the results were adjusted for covariates or not.*

*After appending the Crude ORs to Table 3, the table became excessively large. To improve readability and organization, we divided the content into two separate tables: Table 3 and Table 4. Subsequently, all following tables were renumbered accordingly.*

*We believe these changes improve the clarity and robustness of our findings. Thank you for your constructive suggestion, which has significantly strengthened the manuscript.*

**Point 3.** The current tables (Table 2, 3, 4, 5) provide Adjusted ORs for confounders such as age, education level, marital status, etc.

Confounders are not the primary exposure variables in this study, so providing Adjusted OR for them is not appropriate. The tables should report OR values only for the key independent variables.

**Response 3:** *Thank you for your insightful comment regarding the presentation of Adjusted Odds Ratios (ORs)* *for confounders in the tables. We agree that confounders such as age, education level, marital status, etc., are not the primary exposure variables in this study, and reporting Adjusted ORs for these variables may not be appropriate.*

*In response to your feedback, we have revised Table 2 to remove the Adjusted ORs for confounders. Also, we have deleted the accompanying sentences in the text that explained the Adjusted ORs for confounders. The tables now focus solely on reporting aOR values for the key independent variables, as recommended.*

*We divided the participants by each confounder for Table 3 and 4 (previously Table 3). We conducted multiple logistic regression analysis between HGS and anxiety in each subgroup. Since these subgroup analyses provided meaningful insights, we have retained them in the manuscript.*

*In Tables 5 and 6 (previously Tables 4 and 5), ORs for confounders were not provided because the confounders were used exclusively to adjust for covariates. Therefore, we have retained Tables 5 and 6 in the manuscript.*

*We believe these changes improve the clarity and relevance of the results presented. Thank you for your valuable suggestion, which has helped enhance the quality of our manuscript.*

***Revised manuscript, line 162-164, page 10, Results: Both before and after adjusting for covariates, the risk of having anxiety was significantly higher among women who had low HGS (crude OR [cOR]: 1.33, 95% CI: 0.67–2.63 in men, cOR: 1.989, 95% CI: 1.23–3.21 in women, adjusted OR [aOR]: 1.22, 95% CI: 0.62–2.43 in men, aOR: 2.17, 95% CI: 1.31–3.61 in women).***

***Revised manuscript, line 172-175, page 11, Results: Both before and after adjusting for covariates, no statistically significant differences were found in any male subgroup, in contrast to female subgroups.***

***Revised manuscript, line 261-265, page 18, Discussion: Although aOR for the college or higher group (aOR: 3.41, 95% CI: 0.70–16.64) was higher than that of the high school or lower group (aOR: 2.06, 95% CI: 1.22–3.47), there was no statistical significance in the college or higher group, while there was statistical significance in the high school or lower group, according to the subgroup analysis for education level after adjusting for covariates (Table 4).***

***Revised manuscript, line 269-271, page 18, Discussion: In addition, we found a positive correlation between the degree of asymmetry and the risk of anxiety both before and after adjusting for covariates (Table 5);***

***Revised manuscript, Table 2,3,4,5,6:***

| **Table 2 Association between handgrip strength and anxiety** | | | | | | | | | | | |
| --- | --- | --- | --- | --- | --- | --- | --- | --- | --- | --- | --- |
| **Variables** | | **Anxiety (adjusted)** | | | | | **Anxiety (unadjusted)** | | | | |
|  |  | **aOR** | **95% CI** | | | **p-value** | **cOR** | **95% CI** | | | **p-value** |
| **Male** | **HGS** |  |  |  |  | 0.5654 |  |  |  |  | 0.4102 |
|  | High | 1 |  |  |  |  | 1 |  |  |  |  |
|  | Low | 1.221 | (0.62 | - | 2.43) |  | 1.33 | (0.67 | - | 2.63) |  |
| **Female** | **HGS** |  |  |  |  | 0.0029 |  |  |  |  | 0.0050 |
|  | High | 1 |  |  |  |  | 1 |  |  |  |  |
|  | Low | 2.173 | (1.31 | - | 3.61) |  | 1.989 | (1.23 | - | 3.21) |  |

| **Table 3 Subgroup analysis of the relationship between handgrip strength and anxiety among male** | | | | | | | | | | | | | |
| --- | --- | --- | --- | --- | --- | --- | --- | --- | --- | --- | --- | --- | --- |
| **Variables** | | **Anxiety** | | | | | | | | | | | |
|  |  | **Adjusted** | | | | | | **Unadjusted** | | | | | |
|  |  | **High HGS** | **Low HGS** | | | | | **High HGS** | **Low HGS** | | | | |
|  |  | **aOR** | **aOR** | **95% CI** | | | **p-value** | **cOR** | **cOR** | **95% CI** | | | **p-value** |
| **Age** | |  |  |  |  |  |  |  |  |  |  |  |  |
|  | 60-69 | 1 | 0.75 | (0.19 | - | 2.88) | 0.6704 | 1 | 0.83 | (0.24 | - | 2.95) | 0.7749 |
|  | 70- | 1 | 1.72 | (0.68 | - | 4.34) | 0.2515 | 1 | 2.02 | (0.88 | - | 4.62) | 0.0950 |
| **Education Level** | |  |  |  |  |  |  |  |  |  |  |  |  |
|  | High school or lower | 1 | 1.58 | (0.78 | - | 3.12) | 0.2101 | 1 | 1.35 | (0.69 | - | 2.64) | 0.3731 |
|  | College or higher | 1 | - | - | - | - | <0.001 | 1 | - | - | - | - | <0.001 |
| **Working state** | |  |  |  |  |  |  |  |  |  |  |  |  |
|  | No | 1 | 1.24 | (0.48 | - | 3.24) | 0.6589 | 1 | 1.54 | (0.60 | - | 3.98) | 0.3715 |
|  | Yes | 1 | 0.86 | (0.28 | - | 2.63) | 0.7834 | 1 | 1.26 | (0.43 | - | 3.67) | 0.6688 |
| **Residence** | |  |  |  |  |  |  |  |  |  |  |  |  |
|  | Metropolitan | 1 | 0.75 | (0.31 | - | 1.80) | 0.5138 | 1 | 0.74 | (0.32 | - | 1.73) | 0.4857 |
|  | Rural | 1 | 3.20 | (1.19 | - | 8.57) | 0.0213 | 1 | 5.11 | (1.63 | - | 15.97) | 0.0054 |
| **Marital status** | |  |  |  |  |  |  |  |  |  |  |  |  |
|  | No | 1 | 0.34 | (0.03 | - | 3.77) | 0.3739 | 1 | 0.72 | (0.12 | - | 4.40) | 0.7154 |
|  | Yes | 1 | 1.42 | (0.66 | - | 3.07) | 0.3717 | 1 | 1.45 | (0.69 | - | 3.06) | 0.3254 |
| **Obesity** | |  |  |  |  |  |  |  |  |  |  |  |  |
|  | Underweight | 1 | - | - | - | - | <0.001 | 1 | 1.10 | (0.05 | - | 23.86) | 0.9503 |
|  | Normal | 1 | 1.63 | (0.72 | - | 3.71) | 0.2381 | 1 | 1.63 | (0.75 | - | 3.55) | 0.2199 |
|  | Obese | 1 | 0.64 | (0.13 | - | 3.20) | 0.5482 | 1 | 0.77 | (0.16 | - | 3.81) | 0.7506 |
| **Drink** | |  |  |  |  |  |  |  |  |  |  |  |  |
|  | No | 1 | 1.58 | (0.45 | - | 5.50) | 0.4704 | 1 | 1.85 | (0.59 | - | 5.81) | 0.2870 |
|  | Yes | 1 | 1.05 | (0.43 | - | 2.61) | 0.9100 | 1 | 0.95 | (0.40 | - | 2.24) | 0.9084 |
| **Sleep duration** | |  |  |  |  |  |  |  |  |  |  |  |  |
|  | More than 6h | 1 | 1.04 | (0.40 | - | 2.72) | 0.9327 | 1 | 1.40 | (0.59 | - | 3.31) | 0.4450 |
|  | Less than 6h | 1 | 1.23 | (0.42 | - | 3.55) | 0.7100 | 1 | 1.19 | (0.36 | - | 3.89) | 0.7741 |
| **Stress** | |  |  |  |  |  |  |  |  |  |  |  |  |
|  | Low | 1 | - | - | - | - | <0.001 | 1 | - | - | - | - | <0.001 |
|  | High | 1 | 1.16 | (0.57 | - | 2.33) | 0.6821 | 1 | 1.25 | (0.60 | - | 2.63) | 0.5532 |
| **Physical Activity** | |  |  |  |  |  |  |  |  |  |  |  |  |
|  | insufficient | 1 | 1.08 | (0.49 | - | 2.39) | 0.8478 | 1 | 1.25 | (0.53 | - | 0.29) | 0.6113 |
|  | sufficient | 1 | 1.89 | (0.58 | - | 6.21) | 0.2904 | 1 | 1.42 | (0.50 | - | 4.07) | 0.5125 |
| **Smoke** | |  |  |  |  |  |  |  |  |  |  |  |  |
|  | non-smoker | 1 | 1.18 | (0.54 | - | 2.56) | 0.6753 | 1 | 1.29 | (0.61 | - | 2.75) | 0.5001 |
|  | smoker | 1 | 1.29 | (0.27 | - | 6.28) | 0.7489 | 1 | 1.36 | (0.33 | - | 5.65) | 0.6664 |

| **Table 4 Subgroup analysis of the relationship between handgrip strength and anxiety among female** | | | | | | | | | | | | | |
| --- | --- | --- | --- | --- | --- | --- | --- | --- | --- | --- | --- | --- | --- |
| **Variables** | | **Anxiety** | | | | | | | | | | | |
|  |  | **Adjusted** | | | | | | **Unadjusted** | | | | | |
|  |  | **High HGS** | **Low HGS** | | | | | **High HGS** | **Low HGS** | | | | |
|  |  | **aOR** | **aOR** | **95% CI** | | | **p-value** | **cOR** | **cOR** | **95% CI** | | | **p-value** |
| **Age** | |  |  |  |  |  |  |  |  |  |  |  |  |
|  | 60-69 | 1 | 2.16 | (1.00 | - | 4.70) | 0.0513 | 1 | 2.05 | (0.98 | - | 4.27) | 0.0553 |
|  | 70- | 1 | 2.20 | (1.01 | - | 4.79) | 0.0467 | 1 | 2.32 | (1.11 | - | 4.85) | 0.0253 |
| **Education Level** | |  |  |  |  |  |  |  |  |  |  |  |  |
|  | High school or lower | 1 | 2.06 | (1.22 | - | 3.47) | 0.0071 | 1 | 2.01 | (1.22 | - | 3.30) | 0.0064 |
|  | College or higher | 1 | 3.41 | (0.70 | - | 16.64) | 0.1279 | 1 | 1.92 | (0.45 | - | 8.18) | 0.3780 |
| **Working state** | |  |  |  |  |  |  |  |  |  |  |  |  |
|  | No | 1 | 2.04 | (1.03 | - | 4.04) | 0.0419 | 1 | 2.00 | (1.07 | - | 3.74) | 0.0312 |
|  | Yes | 1 | 2.99 | (1.11 | - | 8.09) | 0.0312 | 1 | 2.39 | (0.99 | - | 5.72) | 0.0515 |
| **Residence** | |  |  |  |  |  |  |  |  |  |  |  |  |
|  | Metropolitan | 1 | 1.92 | (0.98 | - | 3.78) | 0.0578 | 1 | 1.81 | (1.03 | - | 3.18) | 0.0387 |
|  | Rural | 1 | 2.54 | (0.92 | - | 6.98) | 0.0715 | 1 | 2.30 | (0.96 | - | 5.50) | 0.0610 |
| **Marital status** | |  |  |  |  |  |  |  |  |  |  |  |  |
|  | No | 1 | 1.96 | (0.93 | - | 4.13) | 0.0762 | 1 | 1.61 | (0.79 | - | 3.28) | 0.1876 |
|  | Yes | 1 | 2.48 | (1.16 | - | 5.29) | 0.0192 | 1 | 2.16 | (1.02 | - | 4.58) | 0.0452 |
| **Obesity** | |  |  |  |  |  |  |  |  |  |  |  |  |
|  | Underweight | 1 | - | - | - | - | 0.0010 | 1 | 6.72 | (0.97 | - | 46.47) | 0.0536 |
|  | Normal | 1 | 2.02 | (0.97 | - | 4.21) | 0.0596 | 1 | 1.67 | (0.86 | - | 3.26) | 0.1309 |
|  | Obese | 1 | 2.45 | (0.94 | - | 6.38) | 0.0668 | 1 | 2.00 | (0.87 | - | 4.61) | 0.1015 |
| **Drink** | |  |  |  |  |  |  |  |  |  |  |  |  |
|  | No | 1 | 2.44 | (1.20 | - | 4.95) | 0.0137 | 1 | 1.91 | (1.01 | - | 3.62) | 0.0460 |
|  | Yes | 1 | 1.80 | (0.71 | - | 4.57) | 0.2177 | 1 | 2.16 | (0.93 | - | 4.99) | 0.0714 |
| **Sleep duration** | |  |  |  |  |  |  |  |  |  |  |  |  |
|  | More than 6h | 1 | 2.34 | (1.15 | - | 4.76) | 0.0195 | 1 | 2.13 | (1.07 | - | 4.25) | 0.0316 |
|  | Less than 6h | 1 | 1.94 | (0.91 | - | 4.14) | 0.0878 | 1 | 1.78 | (0.88 | - | 3.58) | 0.1078 |
| **Stress** | |  |  |  |  |  |  |  |  |  |  |  |  |
|  | Low | 1 | 4.83 | (0.94 | - | 24.73) | 0.0585 | 1 | 6.82 | (1.51 | - | 30.91) | 0.0131 |
|  | High | 1 | 2.04 | (1.19 | - | 3.50) | 0.0096 | 1 | 1.96 | (0.26 | - | 2.53) | 0.0123 |
| **Physical Activity** | |  |  |  |  |  |  |  |  |  |  |  |  |
|  | insufficient | 1 | 2.07 | (1.11 | - | 3.89) | 0.0233 | 1 | 1.91 | (1.13 | - | 3.25) | 0.0166 |
|  | sufficient | 1 | 2.88 | (1.21 | - | 6.83) | 0.0169 | 1 | 2.26 | (1.00 | - | 5.10) | 0.0507 |
| **Smoke** | |  |  |  |  |  |  |  |  |  |  |  |  |
|  | non-smoker | 1 | 2.18 | (1.30 | - | 3.65) | 0.0033 | 1 | 2.00 | (1.23 | - | 3.25) | 0.0057 |
|  | smoker | 1 | - | - | - | - | <0.001 | 1 | 1.47 | (0.16 | - | 13.84) | 0.7326 |

| **Table 5 Association between HGS-related factors and anxiety** | | | | | | | | | | | |
| --- | --- | --- | --- | --- | --- | --- | --- | --- | --- | --- | --- |
| **Variables** | | **Anxiety (adjusted)** | | | | | **Anxiety (unadjusted)** | | | | |
|  |  | **aOR** | **95% CI** | | | **p-value** | **cOR** | **95% CI** | | | **p-value** |
| **Male** | **HGS** |  |  |  |  |  |  |  |  |  |  |
|  | High | 1 |  |  |  |  | 1 |  |  |  |  |
|  | Low | 1.03 | (0.39 | - | 2.68) | 0.9574 | 1.16 | (0.44 | - | 3.03) | 0.7601 |
|  | Very low | 1.47 | (0.62 | - | 3.53) | 0.3827 | 1.51 | (0.65 | - | 3.53) | 0.3378 |
|  | **HGS asymmetry** |  |  |  |  |  |  |  |  |  |  |
|  | Symmetry | 1 |  |  |  |  | 1 |  |  |  |  |
|  | Moderate asymmetry | 1.52 | (0.75 | - | 3.07) | 0.2407 | 1.44 | (0.76 | - | 2.73) | 2.6390 |
|  | Prominent asymmetry | 0.95 | (0.38 | - | 2.38) | 0.9195 | 1.05 | (0.42 | - | 2.67) | 0.9128 |
| **Female** | **HGS** |  |  |  |  |  |  |  |  |  |  |
|  | High | 1 |  |  |  |  | 1 |  |  |  |  |
|  | Low | 1.46 | (0.69 | - | 3.08) | 0.3176 | 1.35 | (0.64 | - | 2.84) | 0.4218 |
|  | Very low | 3.22 | (1.67 | - | 6.20) | <0.001 | 2.77 | (1.55 | - | 4.94) | <0.001 |
|  | **HGS asymmetry** |  |  |  |  |  |  |  |  |  |  |
|  | Symmetry | 1 |  |  |  |  | 1 |  |  |  |  |
|  | Moderate asymmetry | 1.37 | (0.83 | - | 2.27) | 0.2126 | 1.24 | (0.79 | - | 1.94) | 0.3567 |
|  | Prominent asymmetry | 1.83 | (0.90 | - | 3.72) | 0.0948 | 1.78 | (0.95 | - | 3.35) | 0.0728 |

| **Table 6 Effects of HGS and HGS asymmetry on anxiety** | | | | | | | | | | | | |
| --- | --- | --- | --- | --- | --- | --- | --- | --- | --- | --- | --- | --- |
| **Variables** | | | **Anxiety** | | | | | | | | | |
|  |  |  | **High HGS** | | | | | **Low HGS** | | | | |
|  |  |  | **OR** | **95% CI** | | | **p-value** | **OR** | **95% CI** | | | **p-value** |
| **Male** | **Adjusted** | Symmetry | 1 |  |  |  |  | 1.16 | (0.45 | - | 3.00) | 0.7618 |
|  |  | Moderate asymmetry | 1.59 | (0.70 | - | 3.62) | 0.2696 | 1.44 | (0.44 | - | 4.70) | 0.5441 |
|  |  | Prominent asymmetry | 0.60 | (0.13 | - | 2.87) | 0.5215 | 1.98 | (0.56 | - | 7.00) | 0.2849 |
|  | **Unadjusted** | Symmetry | 1 |  |  |  |  | 1.29 | (0.52 | - | 3.20) | 0.5857 |
|  |  | Moderate asymmetry | 1.52 | (0.70 | - | 3.29) | 0.2923 | 1.49 | (0.55 | - | 4.06) | 0.4302 |
|  |  | Prominent asymmetry | 0.68 | (0.15 | - | 3.14) | 0.6141 | 2.08 | (0.59 | - | 7.31) | 0.2528 |
| **Female** | **Adjusted** | Symmetry | 1 |  |  |  |  | 1.48 | (0.61 | - | 3.60) | 0.3878 |
|  |  | Moderate asymmetry | 1.12 | (0.60 | - | 2.10) | 0.7198 | 2.98 | (1.49 | - | 5.94) | 0.0021 |
|  |  | Prominent asymmetry | 1.37 | (0.49 | - | 3.83) | 0.5502 | 3.16 | (1.37 | - | 7.29) | 0.0075 |
|  | **Unadjusted** | Symmetry | 1 |  |  |  |  | 1.36 | (0.58 | - | 3.18) | 0.4763 |
|  |  | Moderate asymmetry | 1.02 | (0.58 | - | 1.81) | 0.9380 | 2.47 | (1.26 | - | 4.82) | 0.0087 |
|  |  | Prominent asymmetry | 1.29 | (0.50 | - | 3.31) | 0.5927 | 3.01 | (1.28 | - | 7.04) | 0.0116 |
